# Case Report: Combined Small Cell Lung Carcinoma With Pulmonary Adenocarcinoma

**DOI:** 10.3389/fsurg.2022.830849

**Published:** 2022-02-04

**Authors:** Cheng Shen, Guowei Che

**Affiliations:** Department of Thoracic Surgery, West-China Hospital, Sichuan University, Chengdu, China

**Keywords:** combined small cell lung carcinoma, small cell lung carcinoma, surgery, prognosis, adenocarcinoma, case report

## Abstract

**Background:**

Combined small cell lung carcinoma is defined as cancer tissues that mainly contain small cell lung cancer (SCLC) components with non-SCLC (NSCLC) histopathological types. The most common part of NSCLC is squamous cell carcinoma or large cell carcinoma. Combined SCLC (CSCLC) contains adenocarcinoma is extremely rare.

**Case Presentation:**

We reported a case with surgically treated diagnosed as CSCLC with adenocarcinoma in an elderly and we summarized the clinical features of this disease. The patient has remained well for over 2 weeks after the treatment.

**Conclusion:**

There are still few research reports on CSCLC. Since the survival time of patients with advanced CSCLC is shorter than that of simple SCLC, the recommended treatment for CSCLC is early detection and early surgery. In order to facilitate preoperative diagnosis and avoid misdiagnosis of such rare diseases, more cases need to be reported.

## Introduction

The precise definition of combined small cell lung cancer is that the cancer foci are mainly small cell carcinoma with other types of non-small cell, including adenocarcinoma, squamous cell carcinoma, and large cell neuroendocrine carcinoma reported by the WHO (1). Only 13% of primary lung tumors are small cell lung cancer (SCLC). SCLC was first described by Barnard in 1926 as oat celled sarcoma which is the first statement of combined SCLC (CSCLC) in the world. CSCLC is rare in clinical patients, accounting for <1–3.2% of all SCLC (2, 3). We report a case with surgically treated diagnosed as CSCLC with adenocarcinoma in an elderly and we summarized the clinical features of this disease.

## Case Report

**Figure 1:**
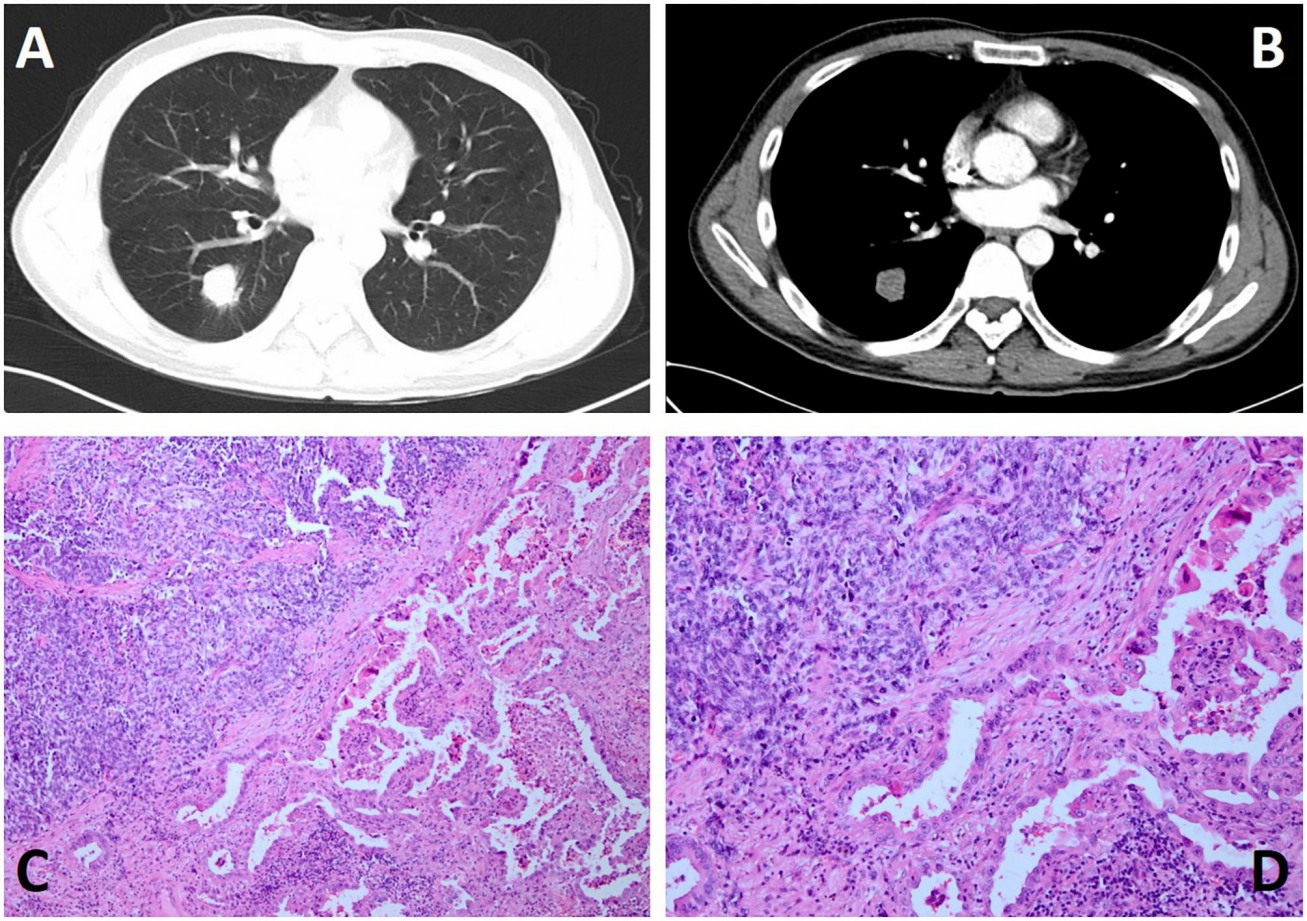
Chest contrast-enhanced CT and histological features and of the case. **(A,B)** Contrast-enhanced CT scan showing a soft tissue, measuring 2.4 cm × 3 cm in size, in the lower lobe of the right lung. **(C,D)** H&E staining reveals the tumor consisted of dual disparate components, which were intermingled in a combined pattern with a definite boundary at low magnification (**C**: ×100 and **D**: ×200).

A 73-year-old man was admitted to our hospital for a routine health check and found a lung nodule by chest X-ray. He had a history of smoking. He had smoked a pack of cigarettes a day for the past 20 years and has now quit smoking for 9 months. When inquiring about the condition at the outpatient clinic, he denied any symptoms such as chest pain, hoarseness, hemoptysis, cough, and difficulty breathing. Physical examination revealed normal breath sounds in both lung fields. There was no obvious abnormality in the laboratory test results. The results of his lung function test and cardiovascular examination were within the normal range. A contrast-enhanced CT scan revealed a soft tissue mass measuring 2.4 cm × 3 cm in the lower lobe of the right lung (Figures 1A,B). Bronchoscopy did not show evidence of pathology. Then, the patient was subjected to lobectomy by utilizing a three-port video-assisted thoracic surgery (VATS). During the operation, we did not find the lesion invaded the adjacent tissue structure. After we cut off the lesion, we used biopsy forceps to remove the tissue from the mass in the specimen plate for rapid freezing pathology, and the pathological diagnosis was small cell lung cancer combined with adenocarcinoma. The postoperative course was uneventful. The patient was followed up for 2 weeks without evidence of recurrence to date.

## Discussion

According to 2004 WHO/International Association for the Study of Lung Cancer (IASLC) classification of lung and pleural tumors, CSCLC is defined as cancer tissues that mainly contain SCLC components with non-SCLC (NSCLC) histopathological types. The most common part of NSCLC is squamous cell carcinoma or large cell carcinoma (4, 5). CSCLC contains adenocarcinoma is extremely rare (6). Only 15 cases including our patient of pulmonary SCLC combined with adenocarcinoma have been reported in 7 studies. As summarized in our Table, the ratio of male and female was 4:1 and the mean age was 68 years (Table 1).

**Table 1:** Clinicopathological features of the CSCLC.

**No**	**Years**	**Age**	**Gender**	**Smoking**	**Location**	**Size (cm)**	**Treatment**	Follow up^*^	**References**
1	2021	79	M	Yes	RLL	3.2x2.4x2.0	VATS	Alive 2 weeks	Ours
2	2009	53	M	Yes	RUL	2.5	thoracotomy+CT+RT	Dead 23 months	Wagner et al. (7)
3	2009	77	M	Yes	LUL	2	thoracotomy+CT	Dead 16 months	Wagner et al. (7)
4	2009	72	F	None	LLL	1.6	thoracotomy+CT+RT	Dead 7 months	Wagner et al. (7)
5	2013	75	F	Not mention	RUL	Not mention	VATS	Alive 16.3 months	Babakoohi et al. (8)
6	2017	60	M	Yes	LLL	2.0x2.0x1.8	thoracotomy	Alive 12 months	Saito et al. (9)
7	2006	71	M	Yes	LLL	4	thoracotomy+CT+RT	Alive 36 months	Iezumi et al. (10)
8	2018	68	F	None	LLL	5	thoracotomy	Not mention	Bai et al. (11)
9	2003	68	M	Not mention	RLL	4	thoracotomy+RT	Dead 1.5 months	Murase et al. (12)
10	2003	71	M	Not mention	LUL	2.5	VATS	Alive 5 months	Murase et al. (12)
11	2003	73	M	Not mention	RLL	4	thoracotomy	Dead 10 days	Murase et al. (12)
12	2007	74	M	Yes	RLL	3.1	VATS	Not mention	Fukui et al. (13)
13	2007	62	F	None	LUL	3.1	VATS	Not mention	Fukui et al. (13)
14	2007	77	M	Yes	LUL	1.5	pneumonectomy	Not mention	Fukui et al. (13)
15	2007	76	M	Yes	RUL	2.8	VATS	Not mention	Fukui et al. (13)

*M, male; F, female; LUL, left upper lobe; LLL, left lower lobe; RUL, right upper lobe; RLL, right lower lobe; VATS, video-assisted thoracic surgery; CT, chemotherapy; RT, radiation therapy;*

* *survival times are post-resection*.

In our analysis, CSCLC is usually asymptomatic and may be diagnosed as an incidental finding and the majority of male patients are smokers, and it illustrates the correlation between CSCLC and smoking. Analyzing the collection cases in our single-center, the patient had no symptoms such as pain or cough, and he was discovered during routine health check-ups. Because tumors are relatively rare, there is currently no definite radiological diagnostic standard for CSCLC. A chest radiograph is the most commonly used imaging method to evaluate lung mass, but it may not be able to distinguish CSCLC from pure SCLC.

Histopathologically, as seen in our case, the tumor lesion is composed of two completely different parts, which can be clearly defined under the microscope. In the SCLC part, the central component is mostly composed of small, uniform, poorly differentiated necrotic cancers (Figures 1C,D), while the other half is considered to be adenocarcinoma with papillary and acinar features. Immunohistochemically, in the pulmonary tumor, TTF-1 vimentin and pancytokeratin were strongly expressed in 75% of the small cell carcinoma cells, and 20% of the small cell carcinoma cells strongly expressed CD56, synaptophysin, and S100 protein (10, 11). As shown in Bai et al. (11) report, The components of SCLC are positive for synaptophysin (Syn), chromogranin A (CgA), and nerve cell adhesion molecule 1 (CD56), and have high proliferative activity by Ki-67 antigen immunostaining, while the adenocarcinoma area had low Ki-67 proliferation activity and was negative to others.

In addition to the analysis of gross specimen morphology and pathology under a microscope, clonality analysis of each component is also useful in CSCLC. In the research of Wagner et al. (7), using loss of heterozygosity (LOH) analysis included 3 and 17 p in the separate components of the lesion in each patient. In the study of Case 3, the alleles changing is inconsistent both in SCLC and NSCLC components. The alleles in SCLC are almost completely lost, while the number of the same alleles is greatly reduced in NSCLC. In case 2 in Table 1, LOH is demonstrated only in the SCLC component. The point mutations in the p53 gene and LOH of chromosome 3p in each component were examined and the results showed that the small cell carcinoma and squamous cell carcinoma components in CSCLC are clone-related, but the adenocarcinoma component comes from a single clone and has no obvious correlation with small cell carcinoma (12).

Although small cell lung cancer is highly sensitive to chemotherapy and radiotherapy, most patients will eventually develop multiple organs metastasis of lung cancer cells. Surgical resection is only for patients with early-stage and no lymph node metastasis. The research suggests that the combination of etoposide and cisplatin is the traditional first-line treatment strategy for SCLC (9). In recent years, with the improvement of technology and detection methods, CSCLC has also been increasingly recognized by more surgeons. The role of surgery in the treatment of CSCLC is increasingly valued (6). For patients with no metastasis to the lymph node, lobectomy with systemic hilar and mediastinal lymphadenectomy are preferred (14). We can see from the Table 1 that the surgery was performed in all patients with video-assisted thoracic surgery (VATS) in six cases or lobectomy by standard thoracotomy in nine patients. Almost all patients with small cell lung cancer have a tendency to spread throughout the body. Therefore, combined chemotherapy and chest radiotherapy are the main treatments for this disease (15, 16). In Table 1, four patients underwent chemotherapy and three of them got radiation therapy. In case 7, reported by Iezumi et al. (10), CSCLC patients received a combined chemotherapy regimen of cisplatin and irinotecan hydrochloride and received radiotherapy for metastasis and recurrence in the forearm. For CSCLC patients with EGFR mutations, use currently mature epidermal growth factor receptor (EGFR) tyrosine kinase inhibitors and other drug-related molecular targeted therapies to exert anti-tumor activity (17, 18).

Some researchers believe that pure SCLC and CSCLC have similar clinical manifestations and that the operation outcomes of both in a limited stage are similar. At present, the clinical characteristics and clinical data of these patients have been published (8, 13, 19, 20). In one study, 5-year survival of patients with CSCLC was only 15.9% (20). They collected the clinical data of CSCLC patients and pure SCLC patients and made detailed comparisons. The final data showed that the prognosis of CSCLC patients and SCLC patients were similar, and there was no statistically significant difference. However, the rate of surgery for CSCLC was much higher compared with pure SCLC. For those advanced patients who did not undergo surgery but received equivalent chemotherapy, the overall survival rate (OS) of CSCLC patients is lower than that of pure SCLC patients (13). Hage et al. (19) also reported that surgical resection in selected patients with pretreatment clinical stage I combined and pure SCLC can be curative or offer long-term survival. However, The OS of patients with pure SCLC is significantly lower than that of patients with CSCLC in Babakoohi et al. (8) research. In their study, the 5-year survival rate of CSCLC patients with stage I or stage II was very good, almost reaching 100%, which is in contrast with the previous study by Hage et al. There are several limitations in this report. Firstly, the short-term follow-up is not enough to fully explain the prognosis in our patient. Secondly, the number of studies included and the simple scale were relatively small.

In short, there are still few research reports on CSCLC. Since the survival time of patients with advanced CSCLC is shorter than that of simple SCLC, the recommended treatment for CSCLC is early detection and early surgery. Surgical resection can only be performed in a small number of patients with conditions, whose can be cured or can obtain long-term survival from preoperative evaluation. In order to facilitate preoperative diagnosis and avoid misdiagnosis of such rare diseases, more cases need to be reported.

## Data Availability Statement

The original contributions presented in the study are included in the article/supplementary material, further inquiries can be directed to the corresponding author/s.

## Ethics Statement

Written informed consent was obtained from the individual(s) for the publication of any potentially identifiable images or data included in this article.

## Author Contributions

CS was involved in drafting the manuscript. GC designed and revised the manuscript. All authors have read and approved the final manuscript.

## Funding

The Science and Technology Project of the Health Planning Committee of Sichuan (No. 19PJ242), Sichuan Province Science and Technology Support Program (No. 2020JDKP0023), and Chengdu science and technology Support Program (No. 2019-YFYF-00090-SN).

## Conflict of Interest

The authors declare that the research was conducted in the absence of any commercial or financial relationships that could be construed as a potential conflict of interest.

## Publisher's Note

All claims expressed in this article are solely those of the authors and do not necessarily represent those of their affiliated organizations, or those of the publisher, the editors and the reviewers. Any product that may be evaluated in this article, or claim that may be made by its manufacturer, is not guaranteed or endorsed by the publisher.
